# Smartphone Alcohol Use Disorder Recovery Apps: Cross-sectional Survey of Behavioral Intention to Use

**DOI:** 10.2196/33493

**Published:** 2022-04-01

**Authors:** Rijuta Menon, Julien Meyer, Pria Nippak, Housne Begum

**Affiliations:** 1 School of Health Services Management Ted Rogers School of Management Ryerson University Toronto, ON Canada

**Keywords:** mobile health, alcohol use disorder, disease management, mobile apps, Unified Theory of Acceptance and Use of Technology

## Abstract

**Background:**

Alcohol use disorder (AUD) carries a huge health and economic cost to society. Effective interventions exist but numerous challenges limit their adoption, especially in a pandemic context. AUD recovery apps (AUDRA) have emerged as a potential complement to in-person interventions. They are easy to access and show promising results in terms of efficacy. However, they rely on individual adoption decisions and remain underused.

**Objective:**

The aim of this survey study is to explore the beliefs that determine the intention to use AUDRA.

**Methods:**

We conducted a cross-sectional survey study of people with AUD. We used the Unified Theory of Acceptance and Use of Technology, which predicts use and behavioral intention to use based on performance expectancy, effort expectancy, social influence, and facilitating conditions. Participants were recruited directly from 2 sources; first, respondents at addiction treatment facilities in Ontario, Canada, were contacted in person, and they filled a paper form; second, members from AUD recovery support groups on social media were contacted and invited to fill an internet-based survey. The survey was conducted between October 2019 and June 2020.

**Results:**

The final sample comprised 159 participants (124 involved in the web-based survey and 35 in the paper-based survey) self-identifying somewhat or very much with AUD. Most participants (n=136, 85.5%) were aware of AUDRA and those participants scored higher on performance expectancy, effort expectancy, and social influence. Overall, the model explains 35.4% of the variance in the behavioral intention to use AUDRA and 11.1% of the variance in use. Social influence (*P*=.31), especially for women (*P*=.23), and effort expectancy (*P*=.25) were key antecedents of behavioral intention. Facilitating conditions were not significant overall but were moderated by age (*P*=.23), suggesting that it matters for older participants. Performance expectancy did not predict behavioral intention, which is unlike many other technologies but confirms other findings associated with mobile health (mHealth). Open-ended questions suggest that privacy concerns may significantly influence the use of AUDRA.

**Conclusions:**

This study suggests that unlike many other technologies, the adoption of AUDRA is not mainly determined by utilitarian factors such as performance expectancy. Rather, effort expectancy and social influence play a key role in determining the intention to use AUDRA.

## Introduction

Alcohol causes 3.3 million deaths a year worldwide, close to 6% of all deaths [1]. Many of these deaths are associated with alcohol use disorder (AUD), defined as “a problematic pattern of alcohol use accompanied by clinically significant impairment or distress” [2]. Treatment and engagement with recovery activities, such as brief interventions, motivational enhancements, and cognitive behavior therapies, are integral to avoiding disease progression [3]. They are well accepted and effective. However, they usually require substantial time, money, and resources; moreover, they depend predominantly on the skill of the clinician and can be stigmatizing [3].

With the advent of smartphones, mobile health (mHealth) apps have been developed to address AUD recovery. These apps can provide information and advice on how to address the condition and help users track their behavior. They serve as accessible, widespread, cost-effective, dependable, individualized, and anonymous alternatives or complements to traditional interventions [3]. These apps have also proved invaluable in the context of the COVID-19 pandemic, which has aggravated addiction issues while severely restricting access to in-person support services. In a 2019 literature review on the efficiency of AUD recovery apps (AUDRA), 63% (n=12) of the 19 studies considered found significant evidence of positive outcomes, 32% (n=6) found none, and 5% (n=1) found negative outcomes for some users [1]. Positive outcomes included decreased alcohol consumption, decreased episodes of binge drinking and alcohol-related injuries, and decreased addiction levels. Despite these benefits, evidence from mHealth app studies indeed suggest low adoption rates [4,5], and studies about the acceptance of mental health apps particularly suggest that potential users remain unconvinced of their usefulness.

Technology adoption has been the subject of significant research attention and conceptualization. The Unified Theory of Acceptance and Use of Technology (UTAUT) is a well-established theory of acceptance of consumer technology [6]. It unifies 8 prominent and competing models of user acceptance of new information technologies [6,7]. With UTAUT2, the model was extended from organizational adoption to a consumer use context [7]. This theory is a good predictor of the intention to use mHealth [8,9], but it has not been used yet to investigate beliefs related to AUDRA. This study was designed to investigate the potential factors contributing to AUDRA adoption among people with AUD.

## Methods

### Study Design and Survey Instrument

**Figure 1:**
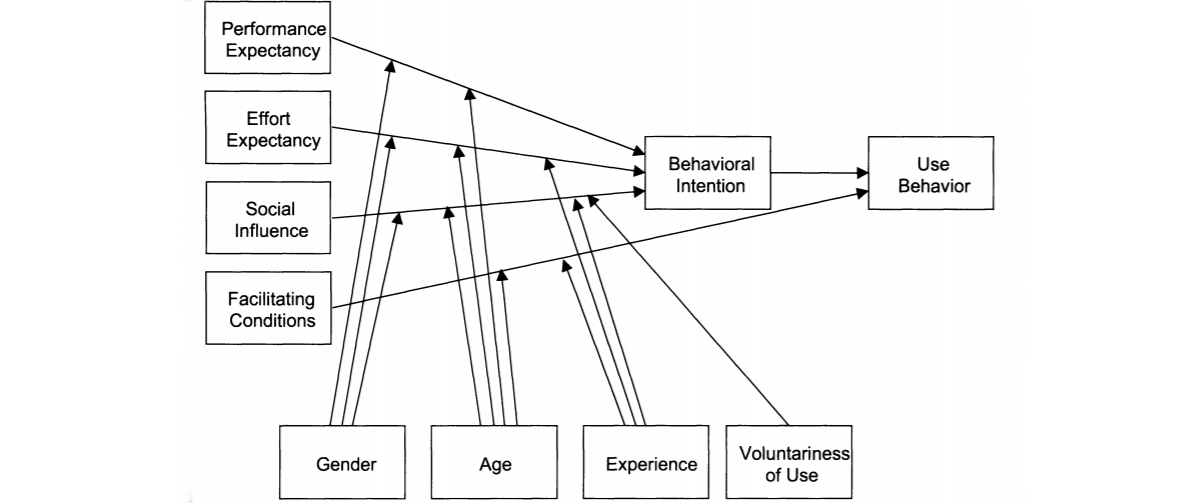
Unified Theory of Acceptance and Use of Technology research model showing the complete theoretical model with the moderating relationships [6].

This study is a cross-sectional survey of nonusers or existing users of AUDRA. The survey covered the factors contributing to the behavioral intention to use smartphone AUD recovery apps among participants (it targeted use of AUDRA in general and not of any specific app). The UTAUT framework and model questionnaire items (Figure 1 and Textbox 1) were adapted to measure the constructs, particularly its operationalizations from UTAUT2. UTAUT predicts that the behavioral intention to use a technology depends on four factors: (1) performance expectancy, defined as the degree to which using a technology will provide benefits to consumers in performing certain activities; (2) effort expectancy, defined as the degree of ease associated with consumers’ use of technology; (3) social influence, defined as the extent to which consumers perceive that important others (eg, family and friends) believe they should use a particular technology; and (4) facilitating conditions, defined as consumers’ perceptions of the resources and support available to perform a behavior [6,7].

The constructs of hedonic motivation, price value, and habit from UTAUT2 were removed*.* They are not applicable to this study as AUDRA are not primarily designed for enjoyment; almost all AUDRA are free on app stores, and AUDRA are still new and rare, which diminish the importance of habit and experience. Age and gender also moderate these relations. Figure 1 shows the theoretical model with the moderating relationships.

The constructs were measured by adapting the 16 corresponding items from UTAUT2 [7] using a 5-point Likert scale ranging from “strongly disagree” to “strongly agree,” except for behavioral intention that had choices “yes,” “no,” or “maybe” and use*,* which used a 6-point Likert scale ranging from “everyday” to “at least once a year” (Textbox 1). A follow-up survey was conducted 6 months later to investigate the subsequent usage behavior. The study was approved by Ryerson’s Research Ethics Board (approval reference number: 2019-277).

**Survey items used for each construct.**

**Performance expectancy**

1. I find/would find Smartphone Alcohol Use Disorder (AUD) recovery apps useful in complementing the daily activities I do to help me recover.

2. Using Smartphone AUD recovery apps helps/would help me learn recovery skills more quickly.

3. Using Smartphone AUD recovery apps helps/would help me increase the effectiveness of activities I do to help me recover.

**Effort expectancy**

4. Learning how to use Smartphone AUD recovery apps is/would be easy for me.

5. My interaction with Smartphone AUD recovery apps is/would be clear and understandable.

6. I find/would find Smartphone AUD recovery apps addiction recovery apps easy to use.

7. It is/would be easy for me to become skillful at using Smartphone AUD recovery apps.

**Social influence**

8. People who are important to me think that I should use Smartphone AUD recovery apps.

9. Caregivers think that I should use Smartphone AUD recovery apps.

10. People who influence my behavior think that I should use Smartphone AUD recovery apps.

11. People whose opinions that I value prefer that I use Smartphone AUD recovery apps.

**Facilitating conditions**

12. I have the resources necessary to use Smartphone AUD recovery apps.

13. I have the knowledge necessary to use Smartphone AUD recovery apps.

14. Smartphone AUD recovery apps are compatible with other technologies I use.

15. I can get help from others to use Smartphone AUD recovery apps.

**Behavioral intention**

16. Do you intend to use or keep using a Smartphone AUD recovery app(s)?

**Use**

17. If you are using a Smartphone app that assists with recovery AUD, how often do you use it?

### Recruitment

Participants were aged 18 years and older, self-identified as having an AUD, and owned a smartphone. Data were collected between October 2019 and June 2020. The survey was offered to participants in 2 modalities. The first was in a pen and paper format, with participants recruited from 6 AUD treatment facilities in Ontario, Canada. Second, an internet-based version of the survey was shared on various English-speaking AUD recovery groups. Participants were offered a gift certificate for their participation. A second follow-up survey was conducted to track usage longitudinally, but it was discarded due to an insufficient response rate. In the partial least squares-structural equation model (PLS-SEM), the minimum sample size is 10 times the number of paths targeting a particular construct. In our study, this means a minimum of 40 respondents [10].

### Statistical Analysis

Internal validity was evaluated using the Cronbach alpha and composite reliability (CR) [11]. Values for the Cronbach alpha and CR are considered satisfactory if they are between 0.7 and 0.9 [12]. Convergent validity was assessed using the outer loadings of the indicators and the values of the average variance extracted (AVE) [11]. To help establish convergent validity on a construct, the outer loadings should be 0.708 or higher and the AVE value must be 0.5 or higher to indicate that the construct explains more than 50% of the variance of its indicators [11].

The heterotrait-monotrait ratio was used to assess the discriminant validity between constructs. When constructs are conceptually more distinct, as is the case with the constructs of UTAUT, a lower conservative threshold of 0.85 is suggested such that values above this threshold indicate a lack of discriminant validity [11].

The results of the survey were analyzed using SPSS Statistics (version 26; IBM Corporation) and SmartPLS 3 (version 3.2.9; SmartPLS GmBH). SPSS Statistics was used for descriptive statistics and chi-square tests were performed to test the associations between variables and differences in the mean scores for variables; their determinants between the 2 groups were assessed using *t* tests at a 95% CI. As the focus of this study was on identifying the antecedents of smartphone AUD recovery app adoption, there were no exclusion criteria in place to exempt the responses of those who did not possess prior knowledge about the existence of these apps. PLS-SEM was used to test the research model (Figure 1) for its reliability, convergent validity, and the discriminant validity of the constructs. The structural model was assessed using *R*² and bootstrapping tests were conducted to examine the statistical significance (taken at 95% CI) of the path coefficients [11]. For the PLS algorithms and bootstrapping calculations, missing data were treated with mean value replacement. SmartPLS 3 was used to test the theoretical model.

The open-ended questions aimed to determine why the participants used or did not use AUDRA. The comments were analyzed quantitatively by themes [13]. Although the low rate of response for these questions did not allow for deriving meaningful statistics, it was sufficient to identify some recurring themes.

## Results

### User Statistics

A total of 1792 surveys were completed. However, most web-based surveys had to be excluded, with 900 excluded for multiple participations, 416 for answering randomly or incompletely, and 317 for not meeting the inclusion criteria (not identifying with AUD or not owning a smartphone) Finally, 159 surveys (124 web-based and 35 paper surveys) could be used.

Table 1 provides the background characteristics of the respondents. The 159 respondents comprised 111 (69.8%) males, 45 (28.3%) females, and 3 (1.9%) individuals who identified themselves as “other” gender. The average age of the respondents was 36 (SD 10.3) years, with a range of 19 to 65 years and mostly between 19 and 39 years (n=117, 73.6%). More than half (n=94, 59.1%) of the participants disclosed their self-identification with AUD as “Very much like me” and the rest (n=65, 40.9%) disclosed it as *“*Somewhat like me.” In terms of prior awareness of AUDRA, 94 participants answered “Very much like me” and 65 participants mentioned “Somewhat like me;” prior awareness of AUDRA was exhibited by 136 (85.5%) participants.

**Table 1:** Sociodemographic characteristics of the respondents (N=159).

Variable		n (%)
**Gender**		
	Male	111 (69.8)
	Female	45 (28.3)
	Other/undisclosed	3 (1.9)
**Age (years)**		
	19-39	117 (73.6)
	40-65	39 (24.5)
	Undisclosed	3 (1.9)
**Self-identification with AUD^a^**		
	Very much like me	94 (59.1)
	Somewhat like me	65 (40.9)
**Prior awareness of AUDRA^b^**		
	Yes	136 (85.5)
	No	23 (14.5)
Total		159 (100)

^a^AUD: alcohol use disorder.

^b^AUDRA: alcohol use disorder recovery app.

### Reliability and Validity of the Constructs

Table 2 describes the reliability and validity of the constructs. Internal validity was evaluated using the Cronbach alpha and CR, with the acceptable range falling between 0.6 and 0.7 [12]. The AVE values for all the constructs, except for facilitating conditions, were above 0.5, thereby indicating convergent validity. Note that the first item, FC1, pertaining to facilitating conditions had to be removed because when FC1 was included along with the other items (FC2, FC3, and FC4), the CR value was very low (0.037). After removing FC1 from facilitating conditions, the CR value improved to 0.621. Therefore, 3 items related to facilitating conditions and all items pertaining to the other constructs were retained.

For the heterotrait-monotrait ratio, all comparisons were well under the recommended threshold of 0.85 and indicated satisfactory discriminant validity between the constructs (Table 2).

Then we compared the constructs to investigate differences between respondents. We compared respondents who identified “somewhat like me” and “very much like me” with AUD, as shown in Table 3. The only significant difference was that the “very much like me” group found it slightly easier to use AUDRA.

Third, we compared respondents based on their prior awareness of AUDRA (Table 4). Respondents aware of AUDRA scored significantly higher on performance expectancy, effort expectancy, and social influence than respondents who had not.

**Table 2:** Construct reliability.

Construct		Cronbach alpha	Average variance extracted	Composite reliability
**Performance expectancy (PE); loading**		.678	0.593	0.812
	PE1; 0.901			
	PE2; 0.714			
	PE3; 0.676			
**Effort expectancy (EE); loading**		.685	0.512	0.806
	EE1; 0.794			
	EE2; 0.650			
	EE3; 0.759			
	EE4; 0.648			
**Social influence (SI); loading**		.766	0.585	0.849
	SI1; 0.720			
	SI2; 0.749			
	SI3; 0.764			
	SI4; 0.824			
**Facilitating conditions (FCs); loading**		.412	0.407	0.621
	FC2; 0.395			
	FC3; 0.976			
	FC4; 0.335			

**Table 3:** Level of identification with alcohol use disorder and participants’ mean scores on Unified Theory of Acceptance and Use of Technology constructs (N=159).

UTAUT^a^ constructs	Self-identification with AUD^b^		
Average out of 5	Very much like me (n=94)	Somewhat like me (n=65)	*P* value
Performance expectancy (3 items)	3.9	4.0	.56
Effort expectancy (4 items)	4.1	3.9	*.03^c^*
Social influence (4 items)	3.7	3.8	.5
Facilitating conditions (4 items)	4.1	4.1	.8
Behavioral intention (1 item)	3.5	3.2	.57
Use behavior (1 item)	2.6	2.6	.93

^a^UTAUT: Unified Theory of Acceptance and Use of Technology.

^b^AUD: alcohol use disorder.

^c^The italicized *P* value is statistically significant.

**Table 4:** Prior awareness of the existence of smartphone alcohol use disorder recovery apps and participants’ mean scores on Unified Theory of Acceptance and Use of Technology constructs (N=159).

UTAUT^a^ constructs	Prior awareness of smartphone AUDRA^b^		
Average out of 5	Yes (n=136)	No (n=23)	*P* value
Performance expectancy (3 items)	4.0	3.6	*.02* ^c^
Effort expectancy (4 items)	4.1	3.7	*.04*
Social influence (4 items)	3.9	3.1	*<.001*
Facilitating conditions (4 items)	4.1	4.0	.45
Behavioral intention (1 item)	2.8	2.7	.74
Use behavior (1 item)	2.7	2.6	.75

^a^UTAUT: Unified Theory of Acceptance and Use of Technology.

^b^AUDRA: alcohol use disorder recovery apps.

^c^The italicized *P* value is statistically significant.

### Structural Model to Identify the Behavioral Factors

**Figure 2:**
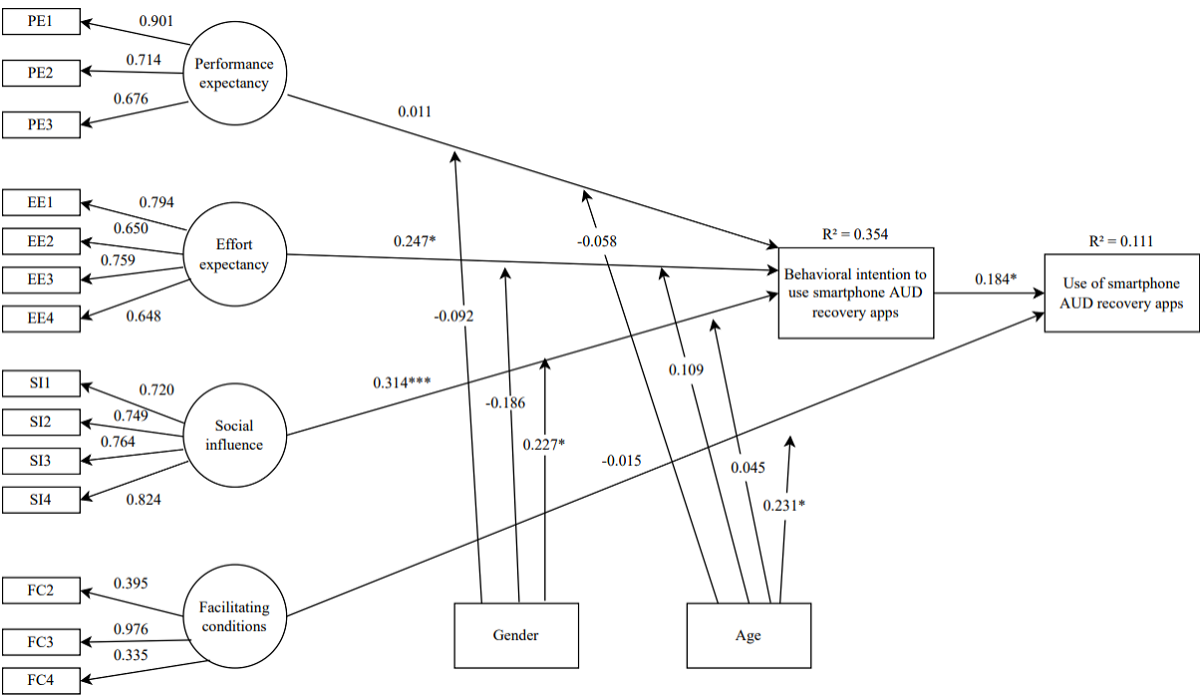
Complete model showing path coefficients and R². Statistical significance of the relationships (path coefficients): *P<.05; **P<.01; ***P<.001. AUD: alcohol use disorder; PE: performance expectancy; EE: effort expectancy; SI: social influence; FC: facilitating conditions.

To analyze the model fit, PLS-SEM was used. Figure 2 shows the path coefficients and the statistical significance of the relationships along with the coefficient of determination or the *R*² value.

Effort expectancy and social influence were significant predictors of behavioral intention to use smartphone AUDRA, which itself predicted use. However, performance expectancy had no effect on behavioral intention. Gender moderated the effect of social influence, meaning that the effect of social influence on behavioral intention was more significant in women than in men. Facilitating conditions had no significant effect on use except for older users who were more likely to be influenced by facilitating conditions. Overall, the model explains 35.4% of the variance in behavioral intention and 11.1% of the variance in use behavior.

### Open-Ended Questions Regarding AUDRA

Open-ended responses provided further insight into participants’ attitudes to AUDRA. Response rates on the 3 questions were between 35% and 67%. Privacy and security concerns were the most frequently given reasons by participants for not wanting to use AUDRA. One respondent stated that “Privacy would be the only issue regarding using an app to help in recovery,“ whereas another pointed out “the potential of data tracking and possibility of using my information for profit.” Other frequently given responses pointed to how “confusing” or “complicated” apps could be. Respondents also expressed their skepticism over the efficacy of such apps in helping them with AUD recovery and noted specific user-unfriendly features, such as too many reminders, notifications, or advertisements: “Pop-ups asking me to rate and/or buy a pro version. Unsolicited communications.” The participants were also dissuaded from potential AUDRA use if there were technical glitches, or “bugginess,” with the apps.

In terms of what would make them want to use AUDRA, respondents asked if these apps would help them with abstinence and prevent relapse. Users often mentioned how a tracking feature (“track my days [without alcohol] and money savings”) helped them. On the contrary, many other users complained about the lack of a tracking feature in the apps they were using. Respondents also frequently cited the ability of apps to connect them with others through social networking features and with local resources, such as if they could “find a meeting close by” and “…Access to events happening through local AA chapte*r*,” as major reasons why they would be encouraged to use the app.

## Discussion

### Principal Results

This study investigated the key antecedents of behavioral intention to use AUDRA among people with AUD. Generally, most of the 159 participants (n=136, 85.5%) were aware of AUDRA. This study confirms the role of effort expectancy and social influence as significant predictors of the intention to use AUDRA, similar to the findings of previous UTAUT studies on mHealth [6,14]. This was confirmed by open-ended answers suggesting that some of the main hurdles to use are technical glitches. However, performance expectancy was not found to significantly predict the intention to use from the final model. This is intriguing because this factor is considered the key determinant of technology usage in general [15-18]. However, it does not appear to apply to mHealth apps [14,19-21]. Other studies have highlighted that despite playing a major role, performance expectancy may not prove salient for mHealth apps when compared to other forms of technology and that effort expectancy plays a much more important role [8].

Facilitating conditions had no direct effect on use, but they were moderated by age. This suggests that facilitating conditions play a more important role as participants age. Other studies conducted with people aged over 60 [20] and 65 years [20,22] have also found a significant influence of facilitating conditions on the use of mHealth apps. Considering that our sample only had 1 participant aged over 60 years, this suggests that the importance of facilitating conditions may start at a younger age.

Participants’ responses to the open-ended questions offer some insights into understanding these results. A major reason given by participants as to why they would not want to use AUDRA was that their privacy, confidentiality, or both could be compromised in any way. This fear has been echoed in many other studies in which respondents cited data privacy concerns as reasons for not using mHealth apps [23-27]. These concerns may have trumped other factors and dampened their intention to use these apps.

Future research should further investigate the factors leading to adoption of mHealth apps, such as concerns regarding privacy. This study also has implications for practitioners. With increased efforts being made to promote the use of AUDRA, designers should first focus on making their apps convenient and easy to use. For app designers, health care professionals, and health care authorities eager to promote the adoption of AUDRA, this study suggests focusing on social influence, ensuring that the use of AUDRA is supported and encouraged by the people who matter to potential users, including their family and general practitioners along with highlighting the positive experiences of other users in their network.

### Limitations

This study has some limitations to be considered when interpreting the findings. We did not have enough respondents in the follow-up survey to measure use longitudinally. In addition, respondents self-identified their AUD status, and we could not verify it; however, previous studies, through test-retest validation, have suggested overall reliability with respect to such self-identification [28] associated with AUD. Many responses also had to be discarded. The gift certificate and the ease of access associated with the internet-based survey on the AUD Facebook groups may have attracted participants who were willing to break the survey rules and may explain the high number of surveys that had to be discarded. Finally, the sample size was relatively small, which comes with associated limitations, notably in terms of statistical power.

### Conclusions

This study found that performance expectancy was not significant in explaining behavioral intention to use AUDRA. Instead, social influence and effort expectancy seem to be the key factors influencing the use of such apps. As apps extend their influence into highly intimate areas of our lives, the beliefs that determine the use of technology may be shifting away from utilitarian factors such as performance. Researchers and app developers alike should keep this in mind and consider the user environment and possibly privacy concerns when developing apps.

## Abbreviations

AUD: alcohol use disorder
AUDRA: alcohol use disorder recovery apps
AVE: average variance extracted
CR: composite reliability
mHealth: mobile health
PLS-SEM: partial least squares-structural equation model
UTAUT: Unified Theory of Acceptance and Use of Technology

## References

[1] Song T, Qian S, Yu P (2019). Mobile health interventions for self-control of unhealthy alcohol use: systematic review. JMIR Mhealth Uhealth.

[2] Kranzler HR, Soyka M (2018). Diagnosis and pharmacotherapy of alcohol use disorder: a review. JAMA.

[3] Fowler LA, Holt SL, Joshi D (2016). Mobile technology-based interventions for adult users of alcohol: a systematic review of the literature. Addict Behav.

[4] Becker S, Miron-Shatz T, Schumacher N, Krocza J, Diamantidis C, Albrecht U (2014). mHealth 2.0: experiences, possibilities, and perspectives. JMIR Mhealth Uhealth.

[5] Vo V, Auroy L, Sarradon-Eck A (2019). Patients' perceptions of mHealth apps: meta-ethnographic review of qualitative studies. JMIR Mhealth Uhealth.

[6] Venkatesh V, Morris MG, Davis GB, Davis FD (2003). User acceptance of information technology: toward a unified view. MIS Quarterly.

[7] Venkatesh V, Thong JYL, Xu X (2012). Consumer acceptance and use of information technology: extending the unified theory of acceptance and use of technology. MIS Quarterly.

[8] Dwivedi YK, Shareef MA, Simintiras AC, Lal B, Weerakkody V (2016). A generalised adoption model for services: a cross-country comparison of mobile health (m-health). Gov Inf Q.

[9] Duarte P, Pinho JC (2019). A mixed methods UTAUT2-based approach to assess mobile health adoption. J Bus Res.

[10] Hair J, Hollingsworth CL, Randolph AB, Chong AYL (2017). An updated and expanded assessment of PLS-SEM in information systems research. IMDS.

[11] Hair JF, Sarstedt M, Ringle CM, Gudergan SP (2017). Advanced Issues in Partial Least Squares Structural Equation Modeling.

[12] Nunnally J, Bernstein I (1994). Psychometric Theory.

[13] O'Cathain A, Thomas KJ (2004). "Any other comments?" Open questions on questionnaires – a bane or a bonus to research?. BMC Med Res Methodol.

[14] Sun Y, Wang N, Guo X, Peng Z (2013). Understanding the acceptance of mobile health services: a comparison and integration of alternative models. Journal of Electronic Commerce Research.

[15] Apolinário-Hagen J, Hennemann S, Fritsche L, Drüge M, Breil B (2019). Determinant factors of public acceptance of stress management apps: survey study. JMIR Ment Health.

[16] Dwivedi YK, Rana NP, Jeyaraj A, Clement M, Williams MD (2017). Re-examining the unified theory of acceptance and use of technology (UTAUT): towards a revised theoretical model. Inf Syst Front.

[17] Taiwo AA, Downe AG (2013). The theory of user acceptance and use of technology (UTAUT): a meta-analytic review of empirical findings. J Theor Appl Inf Technol.

[18] Williams MD, Rana NP, Dwivedi YK (2015). The unified theory of acceptance and use of technology (UTAUT): a literature review. J Enterp Inf Manag.

[19] Carlsson B (2006). Internationalization of innovation systems: a survey of the literature. Res Policy.

[20] Hoque R, Sorwar G (2017). Understanding factors influencing the adoption of mHealth by the elderly: an extension of the UTAUT model. Int J Med Inform.

[21] Nunes A, Limpo T, Castro SL (2019). Acceptance of mobile health applications: examining key determinants and moderators. Front Psychol.

[22] Al-Gahtani SS, Hubona GS, Wang J (2007). Information technology (IT) in Saudi Arabia: culture and the acceptance and use of IT. Inf Manag.

[23] Atienza AA, Zarcadoolas C, Vaughon W, Hughes P, Patel V, Chou W-YS, Pritts J (2015). Consumer attitudes and perceptions on mHealth privacy and security: findings from a mixed-methods study. J Health Commun.

[24] Kao C-K, Liebovitz DM (2017). Consumer mobile health apps: current state, barriers, and future directions. PM & R.

[25] Kotz D, Gunter CA, Kumar S, Weiner JP (2016). Privacy and security in mobile health: a research agenda. Computer.

[26] Krebs P, Duncan DT (2015). Health app use among US mobile phone owners: a national survey. JMIR Mhealth Uhealth.

[27] Zhou L, Bao J, Watzlaf V, Parmanto B (2019). Barriers to and facilitators of the use of mobile health apps from a security perspective: mixed-methods study. JMIR Mhealth Uhealth.

[28] Selin KH (2003). Test-retest reliability of the Alcohol Use Disorder Identification Test in a general population sample. Alcohol Clin Exp Res.

